# Metabolic targeting of ASAH1 in combination with carnosine as a therapeutic strategy for TNBC

**DOI:** 10.1016/j.molmet.2026.102451

**Published:** 2026-09-30

**Authors:** Patrick T. Ball, Ashok Mari, Romi Gupta

**Affiliations:** 1 Department of Biochemistry and Molecular Genetics, The University of Alabama at Birmingham, Birmingham, AL, 35233, USA; 2 O'Neal Comprehensive Cancer Center at The University of Alabama at Birmingham, Birmingham, AL, 35233, USA

**Keywords:** TNBC, ASAH1, Carmofur, Carnosine, Ceranib-2

## Abstract

Triple-negative breast cancer (TNBC) is a highly aggressive breast cancer subtype with limited targeted therapies, leading to higher mortality compared with other breast cancer subtypes. In our previous study we identified N-acyl sphingosine amidohydrolase 1 (ASAH1) to be overexpressed in TNBC cells and demonstrated its role in promoting TNBC growth and progression. ASAH1 is a metabolic enzyme that converts ceramide into sphingosine and free fatty acids, thereby creating a favorable environment for tumor growth. To gain a more comprehensive understanding of the metabolic alterations associated with ASAH1 inhibition, we performed a large-scale metabolomic analysis of TNBC cells expressing ASAH1 shRNAs. Our analysis revealed a significant increase in carnosine levels following ASAH1 inhibition. Subsequent studies demonstrated that exogenous treatment of TNBC cells with carnosine inhibited their growth. Moreover, combining carnosine with ASAH1 inhibitors (carmofur or ceranib-2) resulted in potent synergistic inhibition of TNBC growth. Mechanistically, the combined treatment led to a greater reduction in mitochondrial membrane potential, increased mitochondrial superoxide production and further increased apoptosis. Collectively, these results identify a new metabolism-based combination therapeutic strategy for the effective treatment of TNBC.

## Highlights

• Carnosine exhibits antitumor activity in TNBC and represents a potential therapeutic agent.
• Metabolic co-targeting of ASAH1 with carnosine enhances antitumor efficacy and shows strong translational potential.
• The combination of carnosine and ASAH1 inhibition promote mitochondrial dysfunction and apoptosis in TNBC.
• Co-targeting ASAH1 with carnosine represents a new metabolism-based therapeutic strategy for TNBC.

## 1 Introduction

Triple-negative breast cancer (TNBC) is a subtype of breast cancer that lacks hormone receptors (HR) and human epidermal growth factor receptor 2 (HER2) expression and is associated with metastasis, recurrence, and significantly reduced overall patient survival compared with other subtypes [1,2]. Because of the lack of expression of HER2 and HR markers, there are limited targeted therapeutic options for TNBC, and current treatments primarily rely on standard chemotherapeutic regimens that provide limited clinical benefit [[3], [4], [5]]. Thus, a comprehensive understanding of TNBC is critical for developing more effective therapies.

Cancer cells activate distinct metabolic pathways to support their cellular proliferation, survival, and progression [6,7]. Metabolic deregulation in cancer cells drives tumor development and progression [6,7] and influences the efficacy and outcome of anticancer therapies [8,9]. Deregulation of several metabolic pathways is a key hallmark of cancer cells including TNBC [[10], [11], [12]]. Given the importance of metabolic enzymes and pathways in tumor development and growth, they have become promising therapeutic targets for cancer therapy [8,13,14]. As a result, several metabolic enzyme inhibitors are currently in development, and many are being tested in clinical trials for the treatment of different cancer types [15,16].

In our previous study, we identified the metabolic enzyme ASAH1 to be upregulated in TNBC and demonstrated its role in promoting TNBC growth and progression by modulating the mitogen-activated protein kinase (MAPK) pathway [17]. ASAH1, an acid ceramidase regulates intracellular ceramide level by converting ceramide to sphingosine and free fatty acids, thereby creating a favorable environment for tumor growth [[18], [19], [20]]. We further found that both genetic and pharmacological inhibition of ASAH1 via ASAH1 inhibitor carmofur effectively inhibited TNBC growth. Finally, we demonstrated that and pharmacological co-targeting of ASAH1 (via carmofur) and the MAPK pathway (via trametinib) effectively suppressed TNBC growth [17]. These results established the clinical relevance and translational impact of ASAH1 targeting in TNBC.

In this study, to gain a more comprehensive understanding of the metabolic alterations associated with ASAH1 inhibition, we performed a large-scale metabolomic analysis of ASAH1 knockdown TNBC cells and found significant increase in carnosine levels following ASAH1 inhibition. Carnosine (β-alanyl-l-histidine) is a metabolite that has been shown to exert antitumor effects across multiple cancer types [[21], [22], [23]]. Previous studies have shown that carnosine suppresses glycolytic metabolism and bioenergetic pathways and promotes mitochondrial dysfunction thereby inhibiting cancer cell growth [24,25]. In addition, its role in regulating cell cycle progression and apoptosis, thereby impacting tumor cell survival, has also been reported [21,26]. Consistent with these effects, treatment of cancer cells with carnosine has been observed to suppress tumor growth while exhibiting minimal toxicity toward normal tissues [27], highlighting its therapeutic potential as an anticancer agent.

Our studies demonstrated that exogenous treatment of TNBC cells with carnosine inhibited their growth. More importantly, combining carnosine with the ASAH1 inhibitors (carmofur or ceranib-2) resulted in greater inhibition of TNBC growth than either treatment alone. Mechanistically, the combination treatment disrupted mitochondrial function, as demonstrated by a greater reduction in mitochondrial membrane potential, increased mitochondrial reactive oxygen species production, and enhanced apoptosis. Together, these findings reveal a previously unrecognized link between ASAH1 inhibition, carnosine, mitochondrial dysfunction, and TNBC cell death and identify a new metabolism-based combination therapeutic strategy for the effective treatment of TNBC.

## 2 Results

### 2.1 An unbiased, large-scale metabolomic analysis reveals alterations in several metabolites in ASAH1 knockdown TNBC cells

Because ASAH1 is a metabolic enzyme involved in ceramide/sphingolipid metabolism, we sought to determine whether ASAH1 inhibition induces broader metabolic alterations in TNBC cells. For a comprehensive assessment of metabolic alterations associated with ASAH1 inhibition, we performed a large-scale metabolomic analysis of ASAH1 knockdown TNBC cells. MDA-MB-231 cells expressing either NS shRNA or ASAH1 shRNA were analyzed using capillary electrophoresis time-of-flight mass spectrometry in two modes to detect cationic and anionic metabolites (Figure 1A–C). Our analysis quantified 110 metabolites that were altered in either one or both ASAH1 shRNA-expressing cells as compared to control non-silencing (NS) shRNA expressing cells and were clustered into different metabolic pathways (Figure 1D–E, Supplementary Table 1). Of these metabolites, 64 were significantly altered in both ASAH1 shRNA expressing cell lines compared to control non-silencing (NS) shRNA expressing cells (Figure 1F, Supplementary Table 1). We next mapped these 64 metabolites to different metabolic pathways (Figure 1G) and observed that these metabolites were distributed across various metabolic pathways, including glycolysis, the TCA cycle, the pentose phosphate pathway, nucleotide metabolism, amino acid metabolism and carnosine metabolism (Figure 1G). Collectively, these results demonstrate that suppression of ASAH1 not only alters ceramide/sphingolipid metabolism but is also associated with broader metabolic changes in TNBC cells.

**Figure 1:**
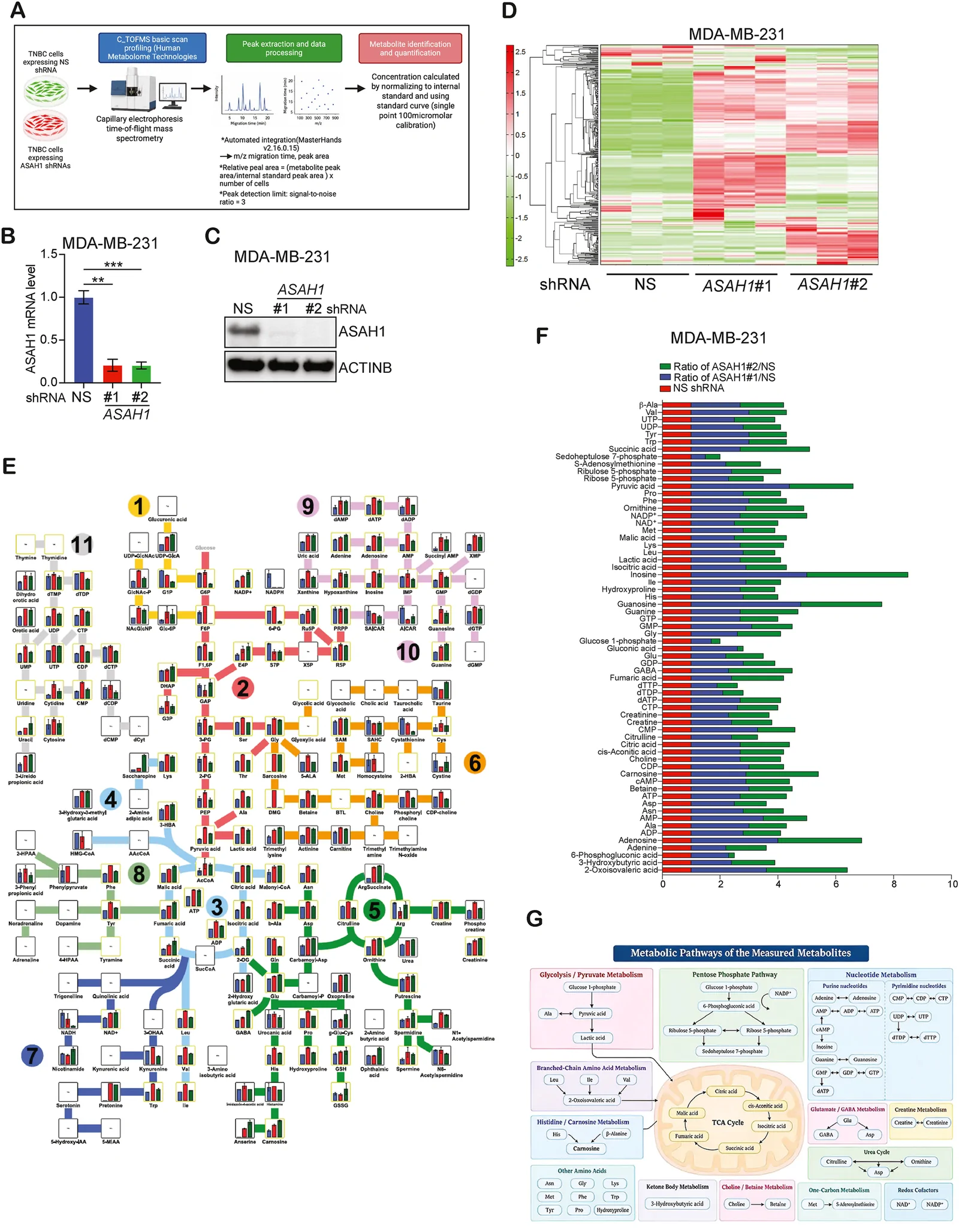
Large Scale metabolomics identifies various metabolites altered in ASAH1 inhibited condition in TNBC cells. **A.** Schematics for large scale metabolomics analysis for MDA-MB-231 expressing either control NS shRNA or two different ASAH1 shRNAs. **B–C.** MDA-MB-231 cells expressing either non-specific (NS) small hairpin RNA (shRNA) or *ASAH1* shRNAs were analyzed by quantitative reverse-transcriptase polymerase chain reaction (RT-qPCR) and immunoblotting to examine *ASAH1* mRNA and protein expression. *ASAH1* mRNA expression level in *ASAH1* shRNA expressing cells relative NS shRNA expressing cells is presented (B). ASAH1 protein expression level in *ASAH1* shRNA expressing cells relative to NS shRNA expressing cells are presented (C). ACTINB was used as the loading control. **D.** Heat map showing altered metabolites in MDA-MB-231 expressing either control NS shRNA or two different *ASAH1* shRNAs. **E**. Among the metabolites that are registered in HMT's standard library, metabolites detected in this study are plotted on the pathway map. The bars/lines represent relative areas of each metabolite in NS shRNA (blue), *ASAH1* #1 shRNA (red), *ASAH1* #2 shRNA (green), respectively. **F**. Bar diagram showing the levels of 64 metabolites that were significantly increased in both *ASAH1* shRNAs expressing MDA-MB-231 cells with respect to control NS shRNA expressing cells. **G.** Pathway analysis for 64 metabolites that were significantly altered is presented. Data represent the mean ± standard error for three biological replicates. ⁎⁎p < 0.01, ⁎⁎⁎p < 0.001.

We then focused on 21 metabolites that were increased by greater than 1.5-fold in both ASAH1 shRNA-expressing MDA-MB-231 cells compared with NS shRNA expressing cells (Figure 2A). Pathway mapping revealed that these metabolites were associated with multiple metabolic pathways (Figure 2B). For example, the detected metabolites included nucleotides and related metabolites (2-oxoisovaleric acid, adenosine, AMP, ATP, cAMP, UMP, and IMP), amino acids and related metabolites (glycine, guanine, lysine, carnosine, ornithine, and β-alanine), and carbohydrate metabolites (glucose, glucose-6-phosphate, and fructose-6-phosphate). Metabolites associated with energy metabolism and the TCA cycle/central carbon metabolism, including pyruvate, lactate, citrate, cis-aconitic acid, malate, and succinate, were also detected. In addition, the analysis identified other metabolites, including inosine, malic acid, NAD^+^, and NADP^+^, as well as the lipid metabolites palmitic acid and stearic acid [28]. Together, these findings demonstrate that ASAH1 knockdown is associated with broad alterations in cellular metabolism and identify metabolites altered that may play an important role in TNBC tumor growth and progression. These results also provide the basis for further evaluating the effects of these metabolites and associated metabolic alterations in TNBC.

**Figure 2:**
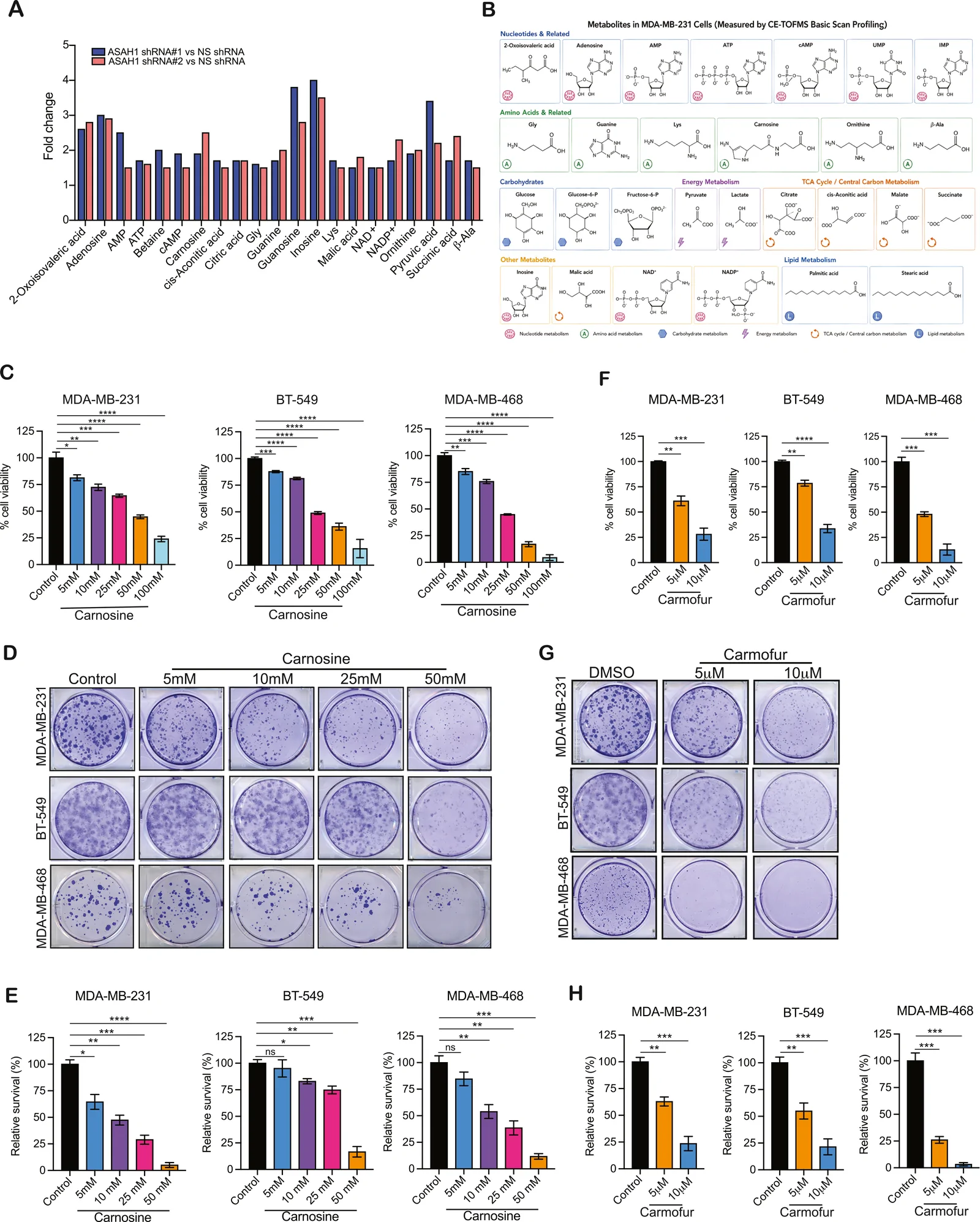
Carnosine treatment suppresses TNBC growth. **A.** Bar diagram showing the levels of 21 altered metabolites that were significantly increased by more than 1.5-fold in both ASAH1 shRNAs expressing cells relative control NS shRNA expressing cells. **B.** Classification of metabolites based on their cellular function. **C.** MDA-MB-231, BT-549 and MDA-MB-468 cells were treated with indicated concentrations of carnosine for 3 days, and survival was assessed 3-(4,5-dimethylthiazol-2-yl)-2,5-diphenyltetrazolium bromide (MTT) assay. Percentage cell viability is plotted in treatment condition relative to control-treated condition. **D.** MDA-MB-231, BT-549 and MDA-MB-468 cells were treated with indicated concentrations of carnosine and analyzed for their ability to survive using clonogenic assays. Representative images are shown. **E.** MDA-MB-231, BT-549 and MDA-MB-468 cells were treated with indicated concentrations of carnosine and were analyzed for their ability to grow using quantitative soft agar assay. Percentage cell survival is plotted in treatment condition relative to control-treated condition. **F.** MDA-MB-231, BT-549 and MDA-MB-468 cells were treated with indicated concentrations of ASAH1 inhibitor carmofur for 3 days, and survival was assessed by 3-(4,5-dimethylthiazol-2-yl)-2,5-diphenyltetrazolium bromide (MTT) assay. Percentage cell viability is plotted in treatment condition relative to DMSO-treated condition. **G.** MDA-MB-231, BT-549 and MDA-MB-468 cells were treated with indicated concentrations of carmofur and analyzed for their ability to survive using clonogenic assays. Representative images are shown. **H.** MDA-MB-231, BT-549 and MDA-MB-468 cells were treated with indicated concentrations of carmofur and were analyzed for their ability to grow using quantitative soft agar assay. Percentage cell survival is plotted in treatment condition relative to control-treated condition. Data represent the mean ± standard error for three biological replicates. ⁎p < 0.05, ⁎⁎p < 0.01, ⁎⁎⁎p < 0.001, ⁎⁎⁎⁎p < 0.0001, ns, not significant.

### 2.2 Carnosine treatment inhibits TNBC growth

We next sought to identify a functionally relevant metabolite among the 21 metabolites that were increased by greater than 1.5-fold in both ASAH1-knockdown conditions. Based on previously published studies, we prioritized carnosine because of its well-established anticancer properties and its potential for therapeutic manipulation [22,29]. Carnosine is a naturally occurring dipeptide with previously reported anticancer properties, including effects on cancer cell proliferation, cellular bioenergetics, oxidative stress, mitochondrial function, and apoptosis [21,24,26,29,30]. Importantly, carnosine can be readily supplemented exogenously, enabling us to directly determine whether increasing carnosine levels could enhance the therapeutic effect of ASAH1 inhibition in TNBC. These studies could also provide the basis for a novel combination treatment strategy that simultaneously targets distinct metabolic vulnerabilities in TNBC cells potentially leading to more effective outcome.

To determine anti-tumorigenic effect of carnosine in our study, we treated multiple TNBC cell lines (MDA-MB-231, MDA-MB-468 and BT-549) with increasing concentrations of carnosine and measured its ability to inhibit the TNBC growth using both short- and long-term cell viability assays. In a short-term MTT survival assay, carnosine effectively inhibited TNBC growth in a concentration-dependent manner (Figure 2C). Similarly, in long-term clonogenic assay, carnosine effectively inhibited the colony formation potential of all tested TNBC cell lines (**Figure D)**. We further tested the effect of carnosine on TNBC cell growth using a quantitative soft agar assay. In this assay also, we observed that carnosine treatment significantly reduced the ability of TNBC cell lines (MDA-MB-231, MDA-MB-468 and BT-549) to form colonies (**Figure E)**. Together, these complementary assays demonstrate that carnosine effectively inhibit the growth of TNBC cells.

### 2.3 Combining carnosine with ASAH1 inhibitors potently inhibited TNBC growth

In our previous study, we demonstrated that the ASAH1 inhibitor carmofur could effectively inhibit TNBC growth [17]. Given our findings that ASAH1 inhibitor carmofur suppresses TNBC growth, we next asked whether combining ASAH1 inhibitor with carnosine could have a greater tumor growth-inhibitory effect on TNBC cells. Before performing the combination studies, we first determined the optimal concentration of carmofur to be used for combination studies by testing its effect on MDA-MB-231, MDA-MB-468 and BT-549 cell viability using MTT, clonogenic, and soft agar assays. We found that carmofur inhibited the growth of TNBC cells in a concentration-dependent manner across all assays (Figure 2F–H). Based on these results, we selected 5 μM carmofur and 10 mM carnosine concentrations, which individually produced only modest effect on the growth inhibition for the combination experiments.

To next evaluated the effect of combination of carnosine and carmofur at the selected doses using MTT, clonogenic and soft agar assay. In these assays, we observed that the combination of carnosine with carmofur, resulted in substantially stronger growth inhibition of TNBC cell lines (MDA-MB-231 and BT-549) (Figure 3A–C) compared with either treatments alone or the vehicle control. To confirm whether the carnosine could effectively work with other ASAH1 inhibitors in addition to carmofur, we performed similar experiments using another ASAH1 inhibitor, ceranib-2. Ceranib-2 has been shown to inhibit ASAH1 and induce apoptosis in cancer cells [[31], [32], [33], [34]]. Consistent with findings with carmofur, we observed that the combination of carnosine and ceranib-2 also resulted a stronger growth inhibition of TNBC cell lines (MDA-MB-231 and BT-549) than either agent alone or the vehicle control across all assays (Figure 3D–F).

**Figure 3:**
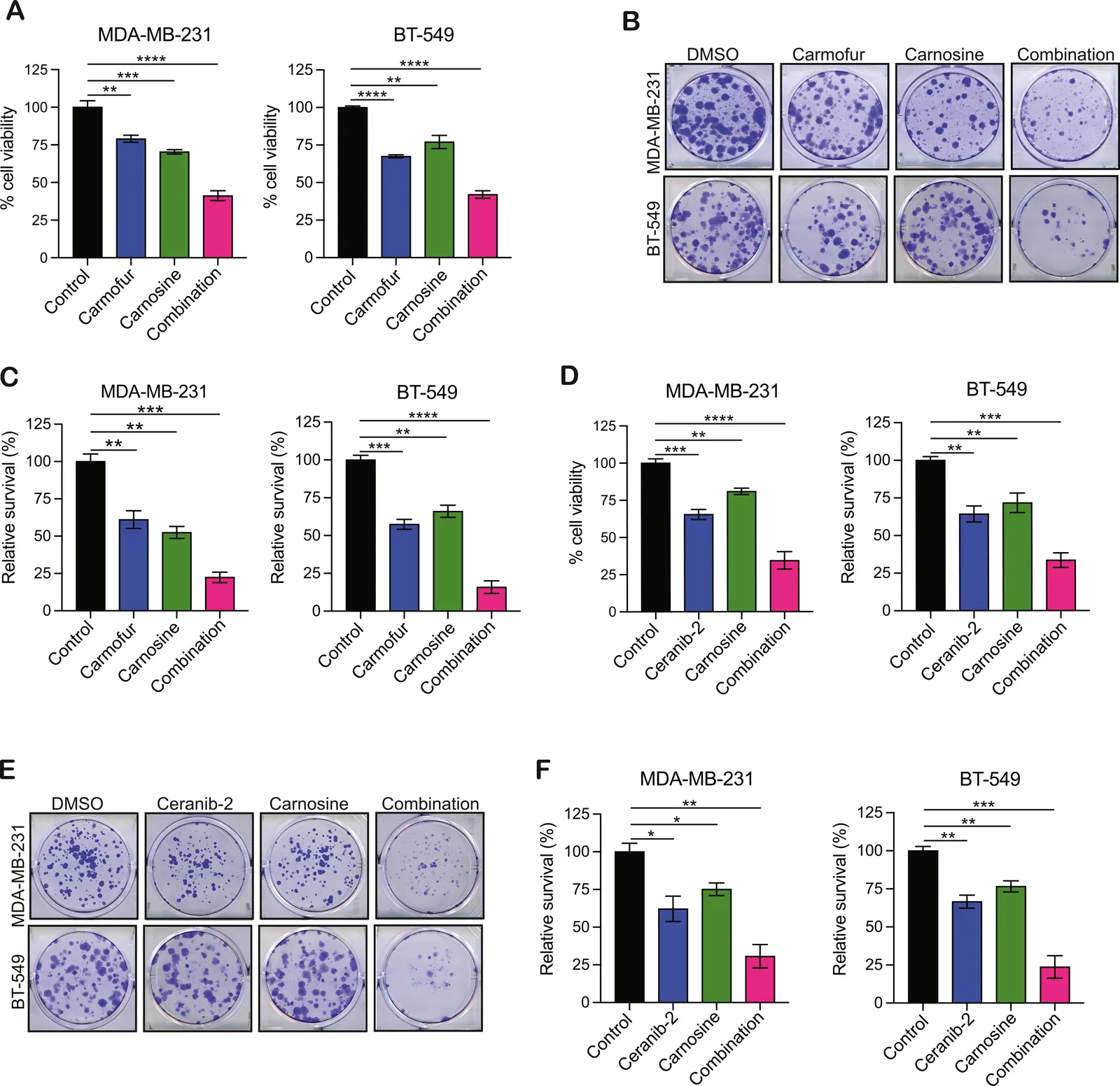
Carnosine potentiates antitumor effect of carmofur in TNBC. **A** and **D.** MDA-MB-231 and BT-549 cells were treated with ASAH1 inhibitor carmofur (5 μM) (**A**) or ASAH1 inhibitor ceranib-2 (5 μM) (**D**) and 10 mM of carnosine for 3 days, and survival was assessed by 3-(4,5-dimethylthiazol-2-yl)-2,5-diphenyltetrazolium bromide (MTT) assay. Percentage cell viability is plotted in treatment condition relative to DMSO-treated condition. **B** and **E.** MDA-MB-231 and BT-549 cells were treated with ASAH1 inhibitor carmofur (5 μM) (**B**) or ASAH1 inhibitor ceranib-2 (5 μM) (**E**) and 10 mM of carnosine and analyzed for their ability to survive using clonogenic assays. Representative images are shown. **C** and **F.** MDA-MB-231 and BT-549 cells were treated with ASAH1 inhibitor carmofur (5 μM) (**C**) or ASAH1 inhibitor ceranib-2 (5 μM) (**F**) and 10 mM of carnosine and were analyzed for their ability to grow using quantitative soft agar assay. Percentage cell survival is plotted in treatment condition relative to control-treated condition. Data represent the mean ± standard error for three biological replicates. ⁎p < 0.05, ⁎⁎p < 0.01, ⁎⁎⁎p < 0.001, ⁎⁎⁎⁎p < 0.0001, ns, not significant. Synergy for combination effect for panel 3 A, 3C, 3D and 3 F was calculated using Bliss independence model with p < 0.05 as significant.

We next performed Bliss independence analysis to determine whether the enhanced effect produced by combining carnosine with ASAH1 inhibitors represent a synergistic interaction. This model is appropriate for our experiments, because a single dose of each agent and their combination were tested in our assays [35]. Bliss independence analysis demonstrated that the antitumor effects observed with the combination of carnosine and ASAH1 inhibitors (carmofur and ceranib-2) were synergistic at the tested concentrations for both TNBC cell lines (Figure 3A,C, 3D, 3F). Together, these results confirm that carnosine can synergize with ASAH1 inhibitors to inhibit TNBC growth, supporting a new and effective potential combination therapy for TNBC.

### 2.4 Combining carnosine with ASAH1 inhibitors induced stronger mitochondrial dysfunction and increased apoptosis

Previous studies have demonstrated that carnosine modulates mitochondrial function and cellular oxidative stress [24,29,[36], [37], [38]], whereas carmofur has been reported to alter sphingolipid metabolism through inhibition of acid ceramidase, a pathway closely linked to mitochondrial dysfunction [39] and apoptosis. Based on these observations, we investigated the effects of carmofur, carnosine, and their combination on mitochondrial dysfunction. To measure mitochondrial dysfunction, we performed two complementary assays. We measured mitochondrial membrane potential and mitochondrial superoxide production. Loss of mitochondrial membrane potential reflects impaired mitochondrial integrity and bioenergetic function, whereas mitochondrial superoxide production indicates mitochondrial oxidative stress [40,41]. Together, these measurements provide complementary evidence of treatment-induced mitochondrial dysfunction.

Before testing the effect of the combination treatment on these two parameters, we first used the positive control carbonyl cyanide m-chlorophenyl hydrazone (CCCP) and tested its effect on mitochondrial depolarization. CCCP is a protonophore/mitochondrial uncoupler that carries protons across the inner mitochondrial membrane and dissipates the proton gradient. Consequently, mitochondrial membrane potential (ΔΨm) decreases (Figure 4A), which can be detected using this assay [42,43]. Consistent with its known activity, we observed that CCCP treatment reduced mitochondrial membrane potential in a concentration-dependent manner in MDA-MB-231 and BT-549 cells (Figure 4B).

**Figure 4:**
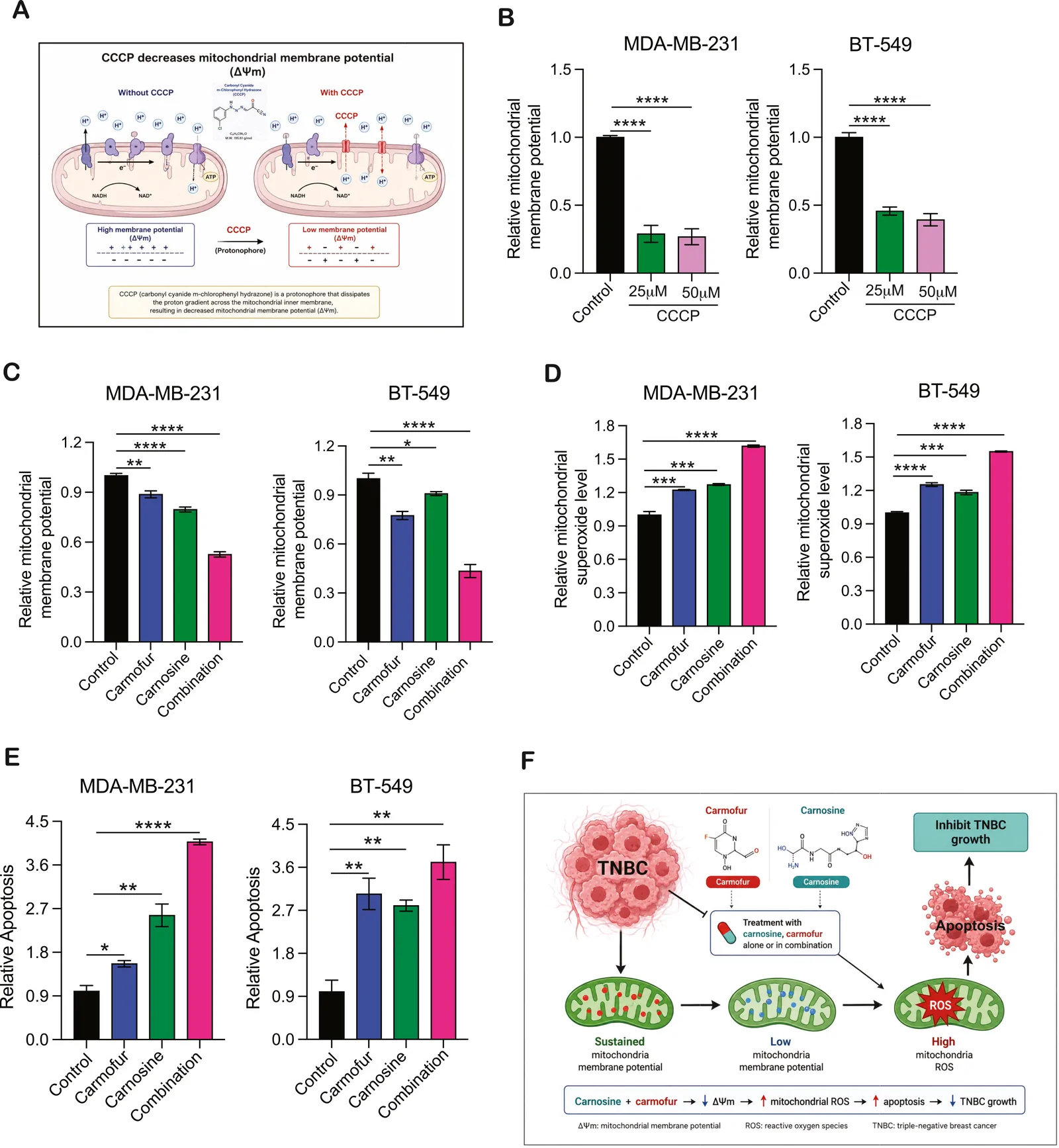
Carnosine in combination with carmofur induce mitochondrial dysfunction and apoptosis in TNBC. **A.** Model showing the effect of CCCP on cells, which is decrease in mitochondrial membrane potential. **B**. Shown TNBC cell lines were either treated with control treated or treated with 25 μM or 50 μM of CCCP for 3 days and mitochondrial membrane potential as measured using Mitochondrial Membrane Potential Assay kit (Cat#13296). Relative mitochondrial membrane potential was plotted in inhibitor treatment condition relative to control treatment. **C**. MDA-MB-231 and BT-549 cells were treated with ASAH1 inhibitor carmofur (5 μM) and 10 mM of carnosine for 3 days, and mitochondrial membrane potential as measured using Mitochondrial Membrane Potential Assay kit (Cat#13296). Relative mitochondrial membrane potential was plotted in treatment condition relative to control treatment. **D**. MDA-MB-231 and BT-549 cells were treated with ASAH1 inhibitor carmofur (5 μM) and 10 mM of carnosine for 3 days, and mitochondrial superoxide as measured using Mitochondrial Superoxide Assay kit (Cat# ab219943). Relative mitochondrial membrane superoxide level was plotted in treatment condition relative to control treatment. **E**. MDA-MB-231 and BT-549 cells were treated with ASAH1 inhibitor carmofur (5 μM) and 10 mM of carnosine for 3 days, and apoptosis was measured using annexin V assay kit (Cat# 556,547). Relative apoptosis was plotted in treatment condition relative to control treatment. **F.** Model summarizing the finding of our study: carnosine in combination with carmofur increases mitochondrial dysfunction (decreased level of mitochondrial membrane potential and increased mitochondrial superoxide formation), which induces apoptosis to suppress TNBC growth. Data represent the mean ± standard error for three biological replicates. ⁎⁎p < 0.01, ⁎⁎⁎p < 0.001, ⁎⁎⁎⁎p < 0.0001, ns: not significant.

We next treated TNBC cells with carnosine and carmofur either alone or in combination and then measured mitochondrial membrane potential. We observed that the combination treatment produced a stronger reduction in mitochondrial membrane potential compared with either the control treatment or the individual treatments (Figure 4C). We further tested the effect of this combination treatment on mitochondrial superoxide production. Consistent with the greater loss of mitochondrial membrane potential, the combination treatment resulted in significantly higher mitochondrial superoxide levels than either individual treatment or the control (Figure 4D). Collectively, these results showed that the combination treatment induced a greater loss of mitochondrial membrane potential, which was associated with increased mitochondrial superoxide levels compared with either the control or individual treatments.

To determine whether the observed mitochondrial dysfunction translated into apoptotic cell death, we performed Annexin V staining to quantify apoptosis. We observed stronger induction of apoptosis with the combination treatment compared with the control or individual treatments (Figure 4E). Collectively, these results indicate that the combination treatment induces significant mitochondrial dysfunction, characterized by decreased mitochondrial membrane potential and increased mitochondrial superoxide production, which leads to stronger apoptosis. These findings provide a mechanistic understanding of the stronger tumor growth-inhibitory effect observed with the combination treatment in TNBC cells (Figure 4F).

## 3 Discussion

Metabolic reprogramming is a hallmark of cancer and contributes to tumor growth, survival, therapeutic resistance, and metastatic progression [44,45]. TNBC exhibits particularly aggressive metabolic adaptations that support its rapid proliferation and poor clinical outcomes [12,46]. Consequently, identifying metabolic vulnerabilities represents an attractive strategy for developing novel therapeutic approaches against TNBC. In the present study, we performed unbiased metabolomic profiling of ASAH1 knockdown TNBC cells and identified several metabolites that were altered upon ASAH1 inhibition. Among the significantly altered metabolites, carnosine emerged as a metabolite that was markedly increased following ASAH1 suppression and with a known potent antitumor activity.

Carnosine (β-alanyl-l-histidine) is a naturally occurring dipeptide that is highly abundant in skeletal muscle and nervous tissue and is recognized for its antioxidant, anti-glycation, and metal-chelating properties [47]. Increasing evidence indicates that carnosine exerts potent antitumor effects across multiple cancer types, including breast, colon, ovarian, and glioblastoma [[21], [22], [23]]. Studies have demonstrated that carnosine inhibits cancer cell proliferation by suppressing glycolytic metabolism, reducing ATP production, and disrupting cellular bioenergetics [24,25,29]. In addition, carnosine induces cell cycle arrest and promotes apoptosis through the modulation of key signaling pathways involved in tumor growth and survival [21]. Carnosine has also been shown to decrease the expression of pro-tumorigenic factors, including vascular endothelial growth factor (VEGF) and cyclin D1, while reducing cancer cell migration and invasion [21]. Furthermore, carnosine has been reported to inhibit the growth of several cancer cell types while exhibiting relatively low toxicity toward normal cells, highlighting its therapeutic potential [27].

To better understand the mechanism underlying increased carnosine levels following ASAH1 suppression, we analyzed our RNA-sequencing and metabolomics data and integrated the two datasets to identify the gene expression changes in ASAH1 knockdown condition that could lead to an increase in carnosine levels. We first found that ASAH1 knockdown increased the expression of SLC36A1, a β-alanine transporter [48,49], accompanied by a concomitant increase in intracellular β-alanine levels in our metabolomics analysis. In addition, ASAH1 knockdown also increased the expression of SLC7A5 and SLC3A2, which form an amino acid transporter complex capable of transporting histidine [50], accompanied by increased intracellular histidine levels observed in our metabolomics analysis. Previous studies have shown that carnosine is synthesized by CARNS1 through the condensation of β-alanine and histidine [21,51]. Based on previous studies and our observations, we postulate that ASAH1 knockdown may promotes carnosine accumulation by increasing the cellular availability of its two precursor amino acids, β-alanine and histidine. Increased availability of these substrates may elevate intracellular carnosine levels under ASAH1 knockdown conditions. Collectively, these findings provide one of the mechanistic explanations for an increase in intracellular carnosine levels upon ASAH1 inhibition. Carnosine act as an antitumor metabolite and thus has the potential to serve as a promising candidate for the development of metabolism-based cancer therapies.

In our previous studies, we demonstrated that ASAH1 is an important driver of TNBC growth and progression and that pharmacological inhibition of ASAH1 with carmofur effectively suppresses TNBC growth. Based on our current finding that ASAH1 suppression increases endogenous carnosine levels, together with the known antitumor properties of carnosine, we reasoned that further increasing carnosine levels through exogenous supplementation might enhance the therapeutic effects of ASAH1 inhibition. Consistent with this hypothesis, combined treatment with carnosine and the ASAH1 inhibitor carmofur produced substantially greater inhibition of TNBC cell viability and colony formation than either agent alone. Importantly, similar effects were observed with ceranib-2, another ASAH1 inhibitor, demonstrating that the enhanced activity was not limited to carmofur. Furthermore, Bliss independence analysis demonstrated a synergistic interaction between carnosine and both ASAH1 inhibitors at the tested concentrations. Together, these findings support the concept that exogenous carnosine supplementation can enhance the antitumor activity of ASAH1 inhibition in TNBC cells.

To further understand the mechanism underlying the enhanced antitumor activity of the combination treatment, we investigated its effects on mitochondrial function. Mitochondrial dysfunction is closely associated with cancer cell death [52,53], however, the effects of combined carmofur and carnosine treatment on mitochondrial function have not previously been characterized in TNBC. We found that the combination treatment caused a greater loss of mitochondrial membrane potential, a marked increase in mitochondrial superoxide production and induced significantly greater apoptosis compared with either treatment alone. These results support a potential link between mitochondrial dysfunction and the enhanced antitumor activity of carmofur combined with carnosine.

Carmofur has been shown to promote the accumulation of pro-apoptotic ceramides [54,55]. In our study carmofur in combination with carnosine further enhanced mitochondrial dysfunction, potentially creating a state of metabolic and mitochondrial stress in TNBC cells resulting in increased apoptotic cell death. This concept is supported by previous studies demonstrating that combined targeting of multiple metabolic pathways can produce stronger antitumor responses than inhibition of a single metabolic pathway [56,57]. Thus, our findings support a model in which ASAH1 inhibition and carnosine supplementation cooperate to enhance metabolic and mitochondrial stress, thereby contributing to increased apoptotic cell death and suppression of TNBC growth.

Our study also has a few limitations. First, our metabolomic analysis measures steady-state metabolite abundance and therefore cannot determine whether changes in individual metabolites result from altered synthesis, degradation, uptake, utilization, or metabolic flux. Therefore, additional isotope-tracing and metabolic flux studies will be required to establish the direction and kinetics of these metabolic alterations. Another limitation of our study is that we evaluated the therapeutic effects of combination of carnosine with ASAH1 inhibition using in vitro assays. These findings provide a strong rationale for testing this combination in vivo to establish their physiological and therapeutic relevance. Additionally, further studies are needed to define the functional significance of other metabolic alterations identified following ASAH1 inhibition to improve overall impact and translational relevance of our study.

Collectively, our findings identify carnosine as an antitumor metabolite that is increased upon ASAH1 inhibition in TNBC cells. More importantly, our results demonstrate that carnosine synergistically enhances the antitumor activity of ASAH1 inhibitors at the tested concentrations, accompanied by increased mitochondrial dysfunction and apoptosis. These results further suggest that carnosine supplementation may enhance the therapeutic efficacy of ASAH1-targeted therapies for cancer treatment. Given the favorable safety profile of carnosine [58,59] and the growing interest in metabolism-targeted cancer therapies [14,16], further investigation of the carnosine–carmofur combination in preclinical TNBC models is warranted. Such studies may ultimately lead to the development of a novel metabolism-based combination therapies for TNBC, for which successful treatment options remain limited.

## 4 Materials and Methods

### 4.1 Cell culture and inhibitors

Triple-negative breast cancer (TNBC) cell lines, including MDA-MB-231 and BT-549 were obtained from the American Type Culture Collection (ATCC). These cell lines were maintained in a humidified atmosphere with 5% CO2 at 37 °C using either Dulbecco's Modified Eagle Medium (Life Technologies, Carlsbad, CA, USA) or Roswell Park Memorial Institute (RPMI)-1640 medium (Life Technologies), as per the recommended guidelines. All media were supplemented with 10% fetal bovine serum (Life Technologies) and 1% penicillin/streptomycin (Life Technologies). Carmofur and carnosine were purchased from Sigma–Aldrich and were prepared for cell culture according to the specifications provided in the datasheet. Information regarding these reagents is listed in the Supplementary Table 2. The treatment conditions are described in the corresponding figure legends.

### 4.2 MTT assay

For this assay, 3 × 10^3^ MDA-MB-231, MDA-MB-468 and BT-549 cells were plated in a 100 μl volume in 96-well plates. After 24 h, the ASAH1 inhibitors (carmofur and ceranib-2), carnosine either alone or in combination were added to the 100 μl media at a range of concentrations as shown in the figure. After 72 h of inhibitor treatment, cell viability was evaluated by adding 20 μl of 5 mg/mL MTT solution dissolved in 1 × PBS to each well, followed by incubation for 2 h in a 37 °C incubator. The MTT solution was gently removed, and 100 μl of dimethyl sulfoxide was added to each well. After mixing by pipetting, absorbance was measured at 590 nm and 630 nm using the BioTek Synergy MX Multi-Format Microplate Reader (Winooski, VT, USA). The average measurement at 630 nm was subtracted from the average measurement at 590 nm, and the relative cell viability at each concentration was plotted with respect to vehicle-treated cells.

### 4.3 Clonogenic assay

Clonogenic assays were performed by seeding 1 × 10^3^ TNBC cells in six-well plates in triplicate. After 24 h, the cells were treated with DMSO, ASAH1 inhibitors (carmofur and ceranib-2), carnosine either alone or in combination were used for clonogenic assays After 2–3 weeks, surviving colonies were fixed in a solution containing 50% methanol and 10% acetic acid and then stained with 0.05% Coomassie brilliant blue R-250 (Sigma–Aldrich). After staining, the plates were scanned with an Epson Perfection V850 Pro Photo Scanner (USA), and representative images were presented.

### 4.4 CytoSelect 96-well quantitative soft agar assay

CytoSelect 96-well Cell Transformation Kit (Cell Biolabs, San Diego, CA, USA) was used to perform quantitative anchorage-independent cell growth assay (soft-agar assay) according to the manufacturer's protocol. Briefly, inhibitor-treated TNBC cells as indicated in shown figure legends cells were seeded in 96-well plates (5 × 10^3^ cells per well) in triplicates. After 7 days of incubation, culture medium was removed by inverting the plate and 50 μL of agar solubilization solution was added and incubated for 1 h at 37 °C. After that 10 μL of the agar mixture and 90 μL CyQuant working solution was added into the 96 well plate and the mixture was incubated for 10 min in the room temperature. The fluorescence was measured at 485/520 nm using a 96-well microplate reader and relative fluorescence units (RFU) were plotted.

### 4.5 Generation of ASAH1 knockdown and validation using qRT-PCR and immunoblotting

Horizon Discovery supplied pLKO.1 lentiviral vector-based shRNAs targeting ASAH1 and a nonspecific (NS) control was used (Supplementary Table 2). Lentiviral particles were generated in 293 T cells using gene-specific or NS shRNA plasmids and packaging plasmids with Effectene Transfection Reagent (Qiagen). TNBC cells were then infected with lentiviral particles and selected with puromycin (0.5–1.5 μg/mL) to generate stable cell lines. After stable generation, total RNA was extracted using TRIzol (Invitrogen) and purified with the RNeasy Mini Kit (Qiagen). cDNA was synthesized using the M-MuLV First Strand cDNA Synthesis Kit (New England Biolabs), followed by qPCR using Power SYBR-Green Master Mix (Applied Biosystems) and gene-specific primers. ACTINB was used for normalization, and primer sequences are listed in Supplementary Table 2. After confirming knockdown at mRNA level ASAH1 level was confirmed using immunoblotting. To do so. Whole-cell lysate from MDA-MB-231 cells expressing either ASAH1 shRNA or control NS shRNA was prepared in IP Lysis Buffer containing protease and phosphatase inhibitors. Protein concentrations were determined by Bradford assay, and equal amounts of protein were separated by 10% or 12% SDS-PAGE and transferred to PVDF membrane. Membrane was blocked with 5% skim milk, incubated with primary antibodies followed by HRP-conjugated secondary antibodies (1:2000), and developed using SuperSignal West Pico or Femto chemiluminescent substrate (Thermo Fisher Scientific). Antibody information is provided in Supplementary Table 2.

### 4.6 Metabolomic analysis

MDA-MB-231 cells expressing either control NS (non-specific) shRNA or ASAH1 shRNA were analyzed for metabolic pathway alterations using the capillary electrophoresis time-of-flight mass spectrometry-based basic scan profiling method of Human Metabolome Technologies (Boston, MA, USA). Cells (1 × 10^6^) for each condition in duplicate were analyzed by this method, and samples were prepared as per the recommendations of Human Metabolome Technologies (Cambridge, MA, USA). For data analysis, peaks detected in capillary electrophoresis time-of-flight mass spectrometry analysis were extracted using automated integration software (MasterHands version 2.16.0.15 developed at Keio University, Tokyo, Japan) to obtain mass/charge ratio (*m*/*z*), migration time and peak area. Peak area was then converted to relative peak area using the following equation: relative peak area = metabolite peak area/internal standard peak area × number of cells. The peak detection limit was determined based on signal-to-noise ratio = 3. Putative metabolites were then assigned from the Human Metabolomic Technologies standard library and known-unknown peak library based on *m*/*z* and migration time. All metabolite concentrations were calculated by normalizing the peak area of each metabolite with respect to the area of the internal standard and by using standard curves, which were obtained by single-point (100 μm) calibrations. The profile of peaks of putative metabolites was represented on metabolic pathway maps using Visualization and Analysis of Networks containing Experimental Data (VANTED) software (http://vanted.ipk-gatersleben.de/).

### 4.7 Mitochondrial Membrane Potential Assay

For this assay, 3 × 10^3^ MDA-MB-231 and BT-549 cells were plated in a 100 μl volume in 96-well plates. After 24 h, carmofur or carnosine either alone or in combination were added as shown in the figure. After 72 h of inhibitor treatment, cell viability was evaluated using Mitochondrial Membrane Potential Assay kit (II) (#13296) as per manufactures protocol. Data presented as the relative difference observed in single and combination treatment as compared to control treated cells.

### 4.8 Mitochondrial Superoxide Assay

For this assay, 3 × 10^3^ MDA-MB-231 and BT-549 cells were plated in a 100 μl volume in 96-well plates. After 24 h, carmofur or carnosine either alone or in combination were added as shown in the figure. After 72 h of inhibitor treatment, mitochondrial superoxide level was evaluated using Mitochondrial Superoxide Assay Kit (Fluorometric) (ab219943) as per manufacture's protocol. Data presented is the relative difference observed in single and combination treatment as compared to control treated cells.

### 4.9 Apoptosis annexin V staining assay

Apoptosis was measured using an annexin V staining kit (BD Pharmingen #556547) according to the manufacturer's instructions. Briefly, 3 × 10^5^ cells/well were seeded in six-well cell culture plates. After the plating, the TNBC cells were treated with carmofur or carnosine either alone or in combination. After the incubation period, the cells were collected, washed twice with 1X PBS, resuspended in 1 × binding buffer, stained with 5 μL FITC-annexin V and 5 μL of PI, and incubated for 15 min in the dark. Next, 400 μL of 1 × binding buffer was added, and the cells were analyzed by flow cytometry using an LSR Fortessa (BD Biosciences). After plating the cells were collected and used for the analysis. Data were analyzed using FlowJo software and relative apoptosis is plotted.

### 4.10 Statistical analysis

All experiments were conducted with at least three biological replicates. Results for individual experiments are expressed as the mean ± standard error of the mean (SEM). Error bars represent SEM. p-values were calculated using two-tailed unpaired Student's *t* tests in the GraphPad Prism software, version 10.0, for Macintosh and p-value <0.05 was considered statistically significant. Drug interaction (synergistic, additive, or simply cumulative) between carmofur and carnosine was calculated using the Bliss independence model [35]. Using this method, Bliss excess scores were calculated independently for each biological replicate, and statistical significance of synergy was determined using a two-sided one-sample t-test comparing the Bliss excess scores with zero. A statistically significant positive Bliss excess (P < 0.05) was considered evidence of a synergistic interaction at the tested concentrations.

## CRediT authorship contribution statement

**Patrick T. Ball:** Data curation. **Ashok Mari:** Data curation. **Romi Gupta:** Writing – review & editing, Writing – original draft, Funding acquisition, Conceptualization.

## Funding

We gratefully acknowledge the following grants from the National Institutes of Health: R03CA292128 (R.G.) and R01CA233481 (R.G.).

## Declaration of competing interest

The authors declare that they have no known competing financial interests or personal relationships that could have appeared to influence the work reported in this paper.

## Data Availability

Data will be made available on request.
