# Supplementary material for: Metabolic targeting of ASAH1 in combination with carnosine as a therapeutic strategy for TNBC
Source: Mol Metab. 2026 Sep 30;114:102451. doi: 10.1016/j.molmet.2026.102451 (PMC13678044; doi:10.1016/j.molmet.2026.102451)
Supplement: Supplementary file 1 [file mmc2.docx]

**Supplementary Table 2:**  Reagents and tools used for this studies.

| Inhibitor | Concentrations | Source |
| --- | --- | --- |
| Carmofur | 5μM, 10μM | Sigma Cat# C1494 |
| Ceranib-2 | 5μM | Sigma Cat# SML0607 |
| Carnosine | 5, 10, 25, 50 and 100 nM | Sigma Cat# C9625 |
| Antibodies |  |  |
| ACTINB | Cell Signaling | Cat#4970 |
| ASAH1 | Sigma-Aldrich | Cat# HPA005468 |
| Commercial assays and kits | | |
| CytoSelect 96-well Cell Transformation Assay Kit | Cell Biolabs | Cat#CBA-130 |
| Mitochondrial Membrane Potential Assay kit | Cell Signaling Technology | Cat#13296 |
| Annexin V staining kit | BD Pharmingen | Cat# 556547 |
| Mitochondrial Superoxide Assay kit | Abcam | Cat# ab219943 |
| Software and Algorithms | | |
| Prism 10.0 | GraphPad | www.graphpad.com/scientific software/prism |
| TNBC cell lines | | |
| MDA-MB-231 | ATCC | HTB-26 |
| BT-549 | ATCC | HTB-122 |
| MDA-MB-468 | ATCC | HTB-132 |
| shRNAs and oligonucleotoides | Clone ID | Catalog number |
| h. ASAH1shRNA #2 | TRCN0000029399 | RHS3979-9596807 |
| h. ASAH1shRNA #2 | TRCN0000029401 | RHS3979-9596809 |
| ACTINB Forward primer | gcatggagtcctgtggcatc |  |
| ACTINB Reverse primer | ttctgcatcctgtcggcaat |  |
| ASAH1 Forward primer | ggaggcaaccagtctgggga |  |
| ASAH1 Reverse primer | ggctggtgcggttcagacac |  |
| Chemicals and Reagents | | |
| DMEM | Sigma-Aldrich | Cat# D5796 |
| RPMI | Sigma-Aldrich | Cat# R8758 |
| Fetal Bovine Serum | GIBCO | Cat# 10437-028 |
| Trypsin-EDTA | GIBCO | Cat# 25200-056 |
| Penicillin-Streptomycin | GIBCO | Cat# 15140-122 |
